# European Court of Justice ruling regarding new genetic engineering methods scientifically justified: a commentary on the biased reporting about the recent ruling

**DOI:** 10.1186/s12302-018-0182-9

**Published:** 2018-12-20

**Authors:** Eva Gelinsky, Angelika Hilbeck

**Affiliations:** 1 Critical Scientists Switzerland (CSS Board), Dändlikerrain 3, 3014 Berne, Switzerland; 2 Semnar/Seed Policy & Science, Tempikon 2, 6283 Baldegg, Switzerland; 3 European Network of Scientists for Social and Environmental Responsibility (ENSSER Board), Marienstrasse 19/20, 10117 Berlin, Germany; 4 Swiss Federal Institute of Technology Zurich, Institute of Integrative Biology, Universitätstrasse 16, 8092 Zurich, Switzerland

**Keywords:** New techniques of genetic engineering, Precautionary principle, European Court of Justice, Bias, Media

## Abstract

In July 2018, the European Court of Justice (Case C-528/16) ruled that organisms obtained by directed mutagenesis techniques are to be regarded as genetically modified organisms (GMOs) within the meaning of Directive 2001/18. The ruling marked the next round of the dispute around agricultural genetic engineering in Europe. Many of the pros and cons presented in this dispute are familiar from the debate around the first generation of genetic engineering techniques. The current wave of enthusiasm for the new genetic engineering methods, with its claim to make good on the failed promises of the previous wave, seems to point more to an admission of failure of the last generation of genetic engineering than to a true change of paradigm. Regulation is being portrayed as a ban on research and use, which is factually incorrect, and the judges of the European Court of Justice are being defamed as espousing “pseudoscience”. Furthermore, this highly polarised position dominates the media reporting of the new techniques and the court’s ruling. Advocates of the new genetic engineering techniques appear to believe that their benefits are so clear that furnishing reliable scientific evidence is unnecessary. Meanwhile, critics who believe that the institution of science is in a serious crisis are on the increase not just due to the cases of obvious documented scientific misconduct by companies and scientists, but also due to the approach of dividing the world into those categorically for or against genetic engineering. In this construct of irreconcilable opposites, differentiations fall by the wayside. This article is a response to this one-sided and biased reporting, which often has the appearance of spin and lacks journalistic ethics that require journalists to report on different positions in a balanced and factual manner instead of taking positions and becoming undeclared advocates themselves.

## Introduction

*“Nothing has been ‘banned’. Interpreting laws that simply recognise the novelty and distinctiveness of different kinds of GM breeding processes, the ECJ is merely offering a consistent framework of interpretation within which continuing healthy reasoned argumentation can be more rigorously played out.”* [1]

On 25 July 2018, the European Court of Justice (ECJ) issued a ruling (Case C-528/16), stating that organisms obtained by directed mutagenesis techniques are to be regarded as genetically modified organisms (GMOs) within the meaning of Directive 2001/18 [2]. These organisms will, therefore, have to be regulated as GMOs, including carrying a GMO label. The ruling also clarified that the exemption of mutagenesis in Annex 1B of the Directive applies only to organisms obtained through the techniques of mutagenesis which have long been used in the conventional breeding and were deemed by the Directive to have a long safety record—which may, however, be the subject of national legislation.

With this judgement, the dispute around agricultural genetic engineering in Europe goes into the next round. In essence, the dispute centres on how the risks arising from these techniques and the organisms developed with it are to be assessed and how they are to be dealt with. Those who advocate an *unregulated* application of these techniques and approval of their products emphasise their precision and safety, and also their potential to enhance “sustainable” agriculture. Those in favour of *regulation* refer to the novelty of the techniques, to the speed with which genetic modifications are now possible, to the increasing indications from research that the techniques could have undesired and likely problematic consequences, as well as to the many unresolved issues, suggesting that *precautions* should be taken with them.

Many of the pros and cons presented are known from the debate around the first generation of genetic engineering techniques. However, what is new is the markedly sharpened tone with which the advocates of the new techniques are speaking out. Furthermore, their highly polarised position dominates the media reporting of the new techniques and of the ECJ judgement.

Some of the central and repeated claims from the advocates of the new techniques, which have been picked up by the media, are listed below—an overview can be found here on a science media outlet [3]:

- The ECJ judgement is said to be unscientific, because it has already been proven that the new genetic engineering techniques are as safe as conventional cultivation methods. A statement in the judgement that is phrased actually rather carefully to the effect that the risks of these new genetic engineering techniques “might” prove to be comparable to the risks occurring with the cultivation and distribution of GMOs by transgenesis (no. 48) is categorically rejected [4–6].
- From this unscientific finding, it is inferred that the judgement is backward-looking and detrimental to progress [6–9].
- Therefore, it is claimed that the innovation ability of Europe as a centre of research and science is essentially jeopardised [4]. It is also claimed that certain necessary innovations, such as an agricultural system that manages with fewer inputs, will not be developed [5, 10, 11].

None of these claims can be backed up with rigorous science. This article is a response to this one-sided and biased reporting, which has the appearance of spin (making biased claims without evidence or with evidence for one side only) and often lacks in journalistic duty of care (give due consideration to different voices also in the science community).

The dispute begins already with different definitions of a GMO (usually a plant). Depending on the position held, conflicting requirements concerning regulation are inferred [12]. Those who advocate an *unregulated* application of both old and new genetic engineering techniques effectively treat GM plants as the sum of its parts, i.e., genes, and only want to subject individual new components to an isolated assessment (reductionistic approach). Those in favour of regulations advocate a comprehensive risk evaluation of the whole GM organism, in which interactions between the GM organism and the environment (including human and animal consumers) are also taken into consideration. Consequently, the argument of those favouring the reductionistic approach is that if no novel transgene constructs are transferred like with the older, conventional genetic engineering techniques, risks would be absent. In contrast, those who favour a comprehensive risk evaluation of the whole GM organism argue that the process of genetic engineering, regardless of what types of molecular scissors are used, carries risks [12], for example, by disrupting or otherwise interfering with the network of genes and their fine-tuned interactions.

Environmental scientists, ecologists, and many medical doctors know that it is often not enough to only react when harm has already been documented and certainty of an impending further danger exists. The establishment of the precautionary principle made it possible to impose regulatory measures even in cases of scientific uncertainty regarding the probability of harm—that is, without definitive proof of harm. This principle is based on the scientific understanding that the complex and often poorly understood interactions between natural processes and technological interventions do not always allow suitable measures to be taken with certainty and in good time to prevent an environmental or human health threat. However, an omission of precautionary measures may lead to irreversible and fatal harm to the environment and human health. In this regard, science has been assigned the key role of providing data, discussing unresolved issues, pointing out uncertainties, and directing attention to surprising, cumulative, synergistic or indirect effects, and their consequences.

In the argumentation of those who advocate an unregulated application of the old and new genetic engineering techniques, this concept of precaution is essentially rejected. They plead for a so-called “evidence-based approach” (also called ‘sound science’) that only justifies state interventions when harm to the environment and health caused by a GMO is conclusively proven [13]. Therefore, it is not a question of a *pre*cautionary (German: Vorsorge) principle, but rather of a *post*cautionary (German: Nachsorge) principle or ‘proof of harm’. This principle is common in USA and the burden of proof as a rule falls upon the victim. Europe counters this with the precautionary principle—a collective civilizational achievement resulting from the bitter lessons learned from past ‘innovations’. The case studies collected in the two volumes of *Late Lessons from Early Warnings* [14, 15] about new technologies and chemical substances with applications that have in retrospect proven fatal, show that too often warnings were ignored or pushed aside until harm to health or the environment was inevitable. In many cases, companies put short-term profit ahead of public safety and hid or ignored the evidence of harm [16, 17]. In other cases, scientists downplayed the risks, sometimes, under pressure from interest groups [16, 17].

Once again, the same points from the debate about the old genetic engineering techniques are being raised. Those who point out risks reject the principle of ‘*post*caution’ and want to perform further investigations *before* any commercial use—yet they are being dismissed, defamed, and attacked. Now, this is also happening to the judges at the ECJ, whose task is simply to interpret the applicable European law, which in turn is based on the precautionary principle. This behavior not only reveals a dubious understanding of science and democracy (a founding principle of which is the independence of the judiciary from private or partisan interests), but also a questionable notion of what the law can and should do in a civil society.

## Precision is not the same as safety

To start with, it is worth looking back to the beginnings of the genetic engineering discussion. It is noticeable that also the old genetic engineering techniques were promoted with claims of ‘naturalness’ and ‘precision’. For example:

“*Genetic engineering is (…) a complementary research tool to identify desirable genes from remotely related taxonomic groups and* ***transfer these genes more quickly and precisely*** *into high*-*yield, high*-*quality crop varieties*.” [18]

“*Molecular techniques now permit the direct and* ***precise introduction of genes*** *from wild relatives, and cellular methods allow screening for the desired phenotype to proceed more efficiently.*” [19]

Back then, it was also inferred that genetic engineering was essentially safe, safer than all forms of conventional breeding, in particular mutagenesis breeding, and, thus, should not be regulated beyond the extent used for variety approval. For example, Sir Robert May, then chief scientific advisor to the UK government, said in a BBC interview in 2000:

“*On the one hand so*-*called GM techniques which in the precise and targeted way bring in a couple of genes that you know what they do and you know where they are is* ***vastly safer, vast, vastly more controlled than this so*-*called conventional breeding*** *that reshuffles about a tenth of the genome*.” [20]

Now, however, even the advocates of deregulation of the new techniques are agreeing with the assessment of the earlier critics, which, at the time, was vilified. This assessment stated that genetic engineering using the older methods was not precise, and for that reason, safety questions should be addressed. Admittedly, this is only stated today to advertise the new genetic engineering techniques as being far safer than the older techniques which, however, are also claimed to be safe.

For example, Prof. Dr. Detlef Weigel of the Max Planck Institute for Developmental Biology in Tübingen writes of the older methods: “*In recent years, it has already been possible to introduce new genes into the plants using genetic engineering methods. They could be genes from other species of plants, but also from completely different organisms, such as bacteria. A* ***disadvantage of this technique until now has been that where the gene ends up in the genetic material cannot be controlled***.” [21].

In comparison, he states regarding the new technique of CRISPR/Cas9:

“*With this method it is… possible to* ***very precisely*** *replace the genes of one species with genes from another variety or a close relative. That is also the aim of conventional breeding. Therefore, genome editing is a way of achieving the same changes as with conventional cultivation, but* ***much faster***.” [21]

However, no reliable evidence that can prove the postulate of ‘speed’ or the postulate of safety has been presented for either the older or the new genetic engineering techniques. On the other hand, there is a need for clarification regarding which process is being accelerated. Admittedly, CRISPR (Clustered Regularly Interspaced Short Palindromic Repeats) makes it possible to produce all the kinds of experimental lines within a short period of time; hence, the high number of publications in this field. However, it is questionable if and how quickly marketable varieties can be developed from these lines that can actually perform in farmers’ fields. We suspect that this will rarely be the case and certainly not exceed the successes of the conventional breeding.

Although it is true that, in comparison to the older genetic engineering techniques, the new genetic engineering techniques such as CRISPR can change genetic material more precisely in specific locations, these interventions may just as much have undesired and unpredictable effects, e.g., on the plant’s metabolism. In the medical field, such off-target effects and its associated risks are not disputed. In fact, recently, in the context of human genetics, scientists and media alike seem to concur with our judgment that these new genetic engineering tools do bear significant risks that ought to be understood better before being widely applied. “*This kind of gene editing* [Crispr/Cas9] *… is still experimental and DNA changes can pass to future generations, potentially with unforeseen side*-*effects. … Many mainstream scientists think it is too unsafe to try…*” [22]. This applies also to plants, if the activity of one enzyme changes, this could cause unintended biochemical reactions. In addition, in plants (as in all organisms), a genetic engineering intervention can lead to plants unintentionally producing modified proteins, potentially resulting in their becoming toxic or allergenic [23–25]. Furthermore, the use of new genetic engineering techniques can have an impact on the environment, for example, when new properties lead to plants having increased survivability (fitness) in comparison to the other plants [26].

## Claims of precision are questionable

In relation to the new genetic engineering techniques such as CRISPR, biotechnologists and science journalists like to make comparisons with text editing programs [27, 28]. They claim that such interventions are no different from editing a text, in which an individual letter is deleted or replaced.

Accordingly, the science editor of the Guardian, Ian Sample, wrote, “*So what is gene editing? Scientists liken it to the find and replace feature used to correct misspelling in documents written on a computer. Instead of fixing words, gene editing rewrites DNA, the biological code that makes up the instruction manuals of living organisms. With gene editing, researchers can disable target genes, correct harmful mutations, and change the activity of specific gene in plants and animals, including humans*.” [29].

Within this comparison, even if it is not factually correct, it is true that we have the tools to precisely cut individual letters from a text and paste them somewhere else in the text. However, someone who does not know the language or the grammar well enough will—notwithstanding this great precision—create nonsense. Yet, nucleotides are not letters and nucleotide sequences are not sentences, but chemical molecules. They follow the rules of biochemistry and not those of human languages or the IT sector. Our understanding of these biochemical rules and the resulting gene functions in interaction with the environment and epigenetic regulation factors can at best be described as limited. The science of epigenetics is quickly developing and is of huge relevance for genetic engineering, yet it is mostly ignored by genetic engineers in the agriculture sector. Therefore, there is a high possibility that unexpected and unpredictable results will be obtained. These results may be good, bad, or trivial, but they are not subject to human control. Thus, the postulate of safety derived from the precision and control narrative is scientifically neither credible nor provable.

In addition, the prematurely declared and exaggerated postulates of precision have already begun to scientifically unravel in recent months. The ECJ judgement is in line with the most recent scientific findings. For example, it only recently became known that the efficiency of CRISPR appears to be associated with the p53 gene, which influences the suppression of tumours in human cells [30, 31]. Scientists can now use mutations in the p53 gene to increase the efficiency of CRISPR. In doing so, they accept that unrepaired DNA (deoxyribonucleic acid) damage in a totally different location in the genetic material leads to an accumulation of unwanted mutations. In further studies, it was conclusively proven that the use of CRISPR can lead to consequences ranging from an unintended modification to the removal of large genome sections [32, 33]. This new evidence shows that the unintended non-target effects of genetic interventions have until now been underestimated. These publications should be understood as an early warning of possible fatal consequences, which have to be thoroughly researched before they can be deemed suitable for mass use. Therefore, in the medical field, this is undisputed, whereas, for plants and the environment, it is disputed. Plants also have a gene with a similar function to p53, the SOG1 gene [34, 35]. It is activated in cases of DNA damage and induces the identification of genes that are responsible for repairing the DNA. It is conceivable that there may be similar correlations between the efficiency of CRISPR and SOG1, but, to date, this remains unknown.

## Extensive modification in organisms possible

New genetic engineering techniques hold the potential to fundamentally modify living organisms. Researchers are in the process of developing the CRISPR methods to the extent that it will be possible to use them repeatedly, simultaneously, or consecutively in the same organism [23, 25, 26, 36]. To date, such extensive interventions are not possible with older methods of genetic engineering. Therefore, we now have to reckon with a much larger number of organisms that have somehow been genetically modified. Against this backdrop, more releases would be possible and they could be accompanied by a multitude of possible, unresearched, unpredictable, and unwanted changes—if the users and applicants are not obligated by law to document such releases and conduct a thorough pre-release risk assessment. Therefore, an even stricter statutory regulation may be warranted than is applied to the older methods of genetic engineering [23].

Even if only individual bases of genetic material are introduced or removed (point mutations) by way of the new genetic engineering techniques, they may greatly modify the organisms. In the worst case, point mutations can mean the difference between life and death: in medicine, there are many examples of hereditary diseases that are based on the smallest modifications of the genetic information [37]. Such interventions could lead to proteins being incorrectly produced or not produced at all. The consequences of such modifications are not necessarily serious; they may even be without consequence. However, nobody can predict this in advance. For precisely that reason, the consequences of a supposedly small intervention, albeit an intervention entailing a new patentable modification, must be thoroughly investigated before the modified organisms are irrevocably released into the environment [25, 26]. The opinion of the judges of the ECJ reflected this scientific assessment. The judges specified how organisms that have been developed using the new tools of genetic engineering are to be regulated. No more, but also no less.

## Old and new overblown promises

For at least 3 decades, massive amounts of tax payers’ money have been invested in the research and funding of biotechnology and genetic engineering [38]. However, this investment is in no way proportionate to the meagre results that have been delivered since then. Both in North America and large parts of South America, the heartlands of the use of agricultural genetic engineering, there is very little (voluntary) regulation [39–41]. ‘Deregulation’ of agricultural biotechnology prevails in those countries—the kind of system that the agricultural biotechnology industry has consistently demanded be replicated in Europe and wherein merely voluntary assessments have to be submitted with no specific requirements for safety data and documentation. Yet even in North and South America, GMO developer companies have not begun to deliver anything close to what it has promised and continues to promise. For more than a quarter of a century, the same GM plant types have dominated: herbicide and insect resistance, integrated into the same industrial crops (soy, corn, cotton, and oilseed rape). The analysis of the biotechnology chapter in the Agriculture at a Crossroads report [42] over 10 years ago is still applicable today—99% of all GM plant types have the aforementioned properties. The few other property types and products of genetic engineering for agricultural applications are distributed over the remaining 1% [43].

Neither has the use of pesticides been reduced nor the yield increased significantly beyond what has been achieved without GM plants [44–48]. In contrast, in some crops, the deployment of pesticides has massively increased. For example, farmers have been driven to an ever-increasing dependence on herbicides to try to control the herbicide-resistant weeds that have accompanied the spread of GM herbicide-tolerant crops [49].

The absence of the promised GMOs with tolerance to drought (except for one claimed drought tolerant event produced by Monsanto) and salinity or with specific ‘consumer traits’ is a problem of genetic engineering [50, 51], not of regulation, and is certainly not down to lack of money or state support (see below). If genetic engineering and its techniques worked in the way that has been promised for decades, then, by now, there ought to be a multitude of custom-made GM products. The fact that this is not the case should be the subject of a serious scientific analysis and self-critical appraisal, and should not be blamed on the bearers of this inconvenient message. For decades, the potential and the promises have remained the same; now, it is time to deliver robust results and not constantly blame others for failing to deliver.

The first plant that was modified using the new genetic engineering techniques, and which is already in cultivation, is (again) a herbicide-tolerant oilseed rape. The new GM plants currently in the companies’ pipelines include a soybean with a modified fatty acid composition, a potato with improved storage capacity at cool temperatures, the so-called waxy maize with a modified starch composition, and a flax, which once again is herbicide-tolerant. Since knock-out plants are produced in approximately 90% of the current applications of CRISPR that are useful only for basic research, such as gene functioning, no commercialisation of the yet again promised ‘super plants’ is to be anticipated in the near future. Properties such as resistance to drought or salt are composed of many different cell components. Plants react to drought, cold, or salt stress with the simultaneous modification of the expression of hundreds of genes. These reactions are adjusted in different parts of the plants to the respective levels of the stress condition [52–54]. Creating stress-tolerant plants in a short time using individual, or even several added, point mutations is a complex, risky, and maybe even impossible task that is also not easier to achieve using the new genetic engineering techniques. Furthermore, the knowledge from various other life science disciplines shows us that organisms are not the sum of their parts and not everything is ‘coded’ in DNA.

## Massive funding, few products, and even less innovation

Over 20 years ago, in the discussions about the conventional genetic engineering and establishing the genetic engineering legislation, warnings were issued concerning the imminent demise of Europe as a global centre for research. It did not happen then, and it will not happen this time around. In the laboratory, biotechnologists can do the work that their freedom of research allows them to do and for which they can acquire funding. In addition, this amounts to a large body of well-funded research. Barely, any other technology and science sector enjoys such a substantial and comprehensive funding basis as biotechnology and, in particular, genetic engineering in all its forms. For decades, the EU and almost all its member states have invested enormous sums of taxpayers’ money in promoting and researching genetic engineering and they continue to do so. Over the years, these sums will have gone into the billions [55]. The genetic engineering laboratories do not suffer from lack of money, as long as they satisfy the scientific and laboratory quality requirements. In addition, this field of research enjoys wide financial and political support from the private sector, philanthrocapitalists (such as the Bill and Melinda Gates Foundation), and large parts of the political sphere, and the EU Commission is certainly not suspected of being critical of genetic engineering—quite the contrary. Even EFSA—the European Food Safety Authority—does what it can to approve GMOs and to accommodate the needs of the users of genetic engineering—to the great annoyance of much of civil society [56, 57]. Other fields of research have never enjoyed such comprehensive and substantial funding and support. In comparison, research into alternative, biological, or agroecological land-use systems and procedures is marginalised, and, to date, has to make do with the proverbial ‘crumbs’, which is also down to the respective structures of research funding [58]. Yet, both agroecological research and conventional breeding do deliver robust data and products for new approaches to a sustainable agriculture. Indeed, they have delivered the very same adapted varieties that genetic engineering has been promising us for decades but has yet to produce [50, 51, 59–61].

## Barely any risk research is worthy of the name

There is very little industry-independent risk research on the possible unexpected, unwanted, and long-term effects of the modification of plants with genetic engineering. There is even less research that has investigated the risks of the new genetic engineering techniques. There is still an enormous disparity between applied studies, which focuses mostly on investigating the possibilities of these techniques and which specific products could be developed, and comprehensive research into the scientific basis and the risks. These risks must be examined using the newest molecular analysis tools. Researchers must consider the interactions between the plants and the environment, as well as generating data on the long-term effects on ecology and the health of consumers. However, application-orientated biotechnology is little interested in the functionality and risks of its subjects; instead, it is too often satisfied when the plants produce the desired property and simply extrapolate their conclusions from the Petri dish to the real world. As an article in Nature put it, “*There is a mentality that as long as it works, we don’t have to understand how or why it works*” [62]. However, questions regarding the biological safety begin where the developers’ interests end. It is unfortunate that, as a rule, this is also where the funding ends.

## Conclusion

*“As the public debates on GMOs have escalated over the last two decades, the roster of partisan (often militant) proponents has grown to include not only industry executives and public relations operatives, but academic basic bioscientists as well.”* [63]

Statements from advocates about the claimed benefits of genetic engineering have been uncritically adopted by the media. However, they fail to differentiate between product development and risk research. The advocates in media and biotechnology science circles appear to believe that the benefits of the techniques are so clear that furnishing reliable evidence is deemed not necessary. Sweeping undocumented claims of safety derived from postulates of control through precision are reminiscent of the early days of the first generation of genetic engineering tools—as are the overblown and similarly undocumented promises of future products. Generations of genetic engineering projects and businesses have come and gone in accelerating tempo, each time raising greater expectations and untold financial fortunes for savvy speculators. However, the evidence continues to point to a failure deep in its very structure and foundation. From the start, risks were considered to be negligible—yet, this assumption was not underpinned by reliable evidence. Furthermore, regulation is being portrayed as a ban on research and use [64], which is factually incorrect, and the ECJ judges, in common with the advocates of regulation, are being defamed as espousing “pseudoscience”. It is debatable how, in this polarised climate, a factual and balanced discussion can take place, both in the scientific community and in society at large.

Critics who believe that the institution of science is in a serious crisis due to this muddled situation are on the increase [63]. This growing disenchantment is not just due to the cases of obvious scientific misconduct by companies and scientists, as was shown, for example, in the case of the “Monsanto Papers” [16], but is also due to the approach of dividing the world into those categorically for or those against genetic engineering. In this construct of irreconcilable opposites, the differentiations fall by the wayside.

To restore integrity of the institution of science, the following reforms should be implemented:

- In new fields that are developing dynamically, such as the new genetic engineering techniques, research should consist of more than just superficial proof that the modified organism allegedly works as desired.
- Those organisms, which, after the intervention, do not have the desired property modification, ought to be studied as well to learn the reasons for failure of expressing the desired property.
- The consequences of a genetic engineering intervention on the cell level and on the level of the whole organism and also the effects that the interaction between the organism and the environment should be investigated in more detail.
- Questions, uncertainties, and unwanted research results should also be openly communicated, as well as disclosure regarding the funding of research and possible restrictions from the funder(s).
- If there is disagreement or lack of conclusiveness, e.g., about the probability of harm occurring, a plausibility check of the available data should be performed. The plausibility should be decided on the basis of scientific criteria that are recognised by the scientific community. Theories or hypotheses must explain a specific phenomenon and be testable, fulfil coherence requirements, and satisfy the principle of organised scepticism (for instance, through peer review). To conduct this check in accordance with scientific criteria, it is necessary to have complete access to the information that has led to the formulation of the scientific theory. The data must be presented in an understandable manner and include any information that does not support the scientific theory. To ensure that the plausibility check is carried out in an unbiased manner and according to scientific criteria, the scientific institutions must be independent.

In theory, all these elements should already be firmly in place and science journalists should critically follow and report about the research findings and their limitations. However, in practice, we are recognising biased reporting where science journalists are becoming advocates of the genetic engineers’ ‘grand hope’ narratives ([65], containing a series of articles conveying enthusiastic stories and narratives of genetic engineers). Instead of rigorous scientific analysis of whether and how the findings emerging from basic science fields such as evolutionary biology, genetics, and epigenetics can be brought in line with the highly reductionistic and deterministic understanding of the DNA-centred paradigm prevailing in the circles of biotechnology engineers, blame is being placed on those who advocate—based on scientific evidence—for stringent regulations and biosafety research. We believe that this development must be resolutely opposed.

## Abbreviations

GMOs: genetically modified organisms
ECJ: European Court of Justice
CRISPR: Clustered Regularly Interspaced Short Palindromic Repeats
DNA: deoxyribonucleic acid
